# Influence of Japan’s 2004 postgraduate training on ophthalmologist location choice, supply and distribution

**DOI:** 10.1186/s12909-018-1147-9

**Published:** 2018-03-27

**Authors:** Rie Sakai-Bizmark, Rei Goto, Shusuke Hiragi, Hiroshi Tamura

**Affiliations:** 1 Department of Social and Behavioral Sciences, Harvard T.H. Chan School of Public Health, Boston, USA; 2 Los Angeles Biomedical Research Institute at Harbor-UCLA Medical Center, Torrance, United States; 3 Department of Pediatrics, David Geffen School of Medicine, University of California, Los Angeles, USA; 4 Graduate School of Business Administration, Keio University, Tokyo, Japan; 5 Keio Business School, Tokyo, Japan; 6 Division of Medical Information Technology and Administration Planning, Kyoto University Hospital, Sakyo-ku, Kyoto City, Kyoto, 606-8507 Japan; 7 Department of Ophthalmology and Visual Sciences, Kyoto University Graduate School of Medicine, Kyoto, Japan

**Keywords:** Japan, Medical residency, Ophthalmologist supply, Postgraduate training program, Quality of life, Patient care, Disparity

## Abstract

**Background:**

Highly-competent patient care is paramount to medicine. Quality training and patient accessibility to physicians with a wide range of specializations is essential. Yet, poor quality of life for physicians cannot be ignored, being detrimental to patient care and leading to personnel leaving the medical profession. In 2004, the Japanese government reformed postgraduate training for medical graduates, adding a 2-year, hands-on rotation through different specialties before the specialization residency was begun. Residents could now choose practice location, but it sparked concerns that physician distribution disparities had been created. Japanese media reported that residents were choosing specialties deemed to offer a higher quality of life, like Ophthalmology or Dermatology, over underserved areas like Obstetrics or Cardiology. To explore the consequences of Japan’s policy efforts, through the residency reform in 2004, to improve physician training, analyzing ophthalmologist supply and distribution in the context of providing the best possible patient care and access while maintaining physician quality of life.

**Methods:**

Using secondary data, we analyzed changes in ophthalmologist supply at the secondary tier of medical care (STM). We applied ordinary least-squares regression models to ophthalmologist density to reflect *community factors* such as residential quality and access to further professional development, to serve as predictors of ophthalmologist supply. Coefficient equality tests examined predictor differences before and after 2004. Similar analyses were conducted for all physicians excluding ophthalmologists (*other physicians*). Ophthalmologist coverage in top and bottom 10% of STMs revealed supply inequalities.

**Results:**

Change in ophthalmologist supply was inversely associated with baseline ophthalmologist density before (*P* < .01) and after (*P* = .01) 2004. Changes in *other physician* supply were not associated with baseline *other physician* density before 2004 (*P* = 0.5), but positively associated after 2004 (*P* < .01). Inequalities between top and bottom 10% of ophthalmologist supply in STMs were large, with best-served areas maintaining roughly five times greater coverage than least-served areas. However, inequalities gradually declined between 1998 and 2012.

**Conclusions:**

Ophthalmologist supply increased both before and after the 2004 reform, yet contrary to media reports, proceeded at a lesser rate than supply increases for *other physicians*. After 2004, geographical disparities decreased for ophthalmologists, while increasing for *other physicians*.

## Backgrounds

Medicine is nothing if not a career dedicated to the needs of patients. High quality physician training is essential. Adequate physician supply, distribution, and accessibility are vital to public health. Yet, inattention to physicians’ quality of life can adversely affect patient care, whether through inadequate personnel supply in the most demanding specialties or in more rural areas, or in the worst case, exodus from the profession. Japan’s government has attempted to leverage improved patient care through training and reduce disparities through its longstanding universal healthcare policy, while conceding to the importance of physician preference in training location and specialty. We looked at some of these competing influences through changes in supply and distribution of ophthalmologists resulting from Japan’s healthcare policy changes.

In 2004, the Japanese Ministry of Health, Labor and Welfare (MHLW) instituted a new postgraduate medical education (PGME) program to improve the quality of medical residency training, which included two major changes [1].

First, a mandatory 2-year general residency program was added through a new national matching system, providing residents with hands-on experience through rotation in several different specialties, such as internal medicine, anesthesiology, etc. Besides helping new doctors develop primary care skills, residents also used that time to consider their specialization. After completion, residents entered their specialty training without the added benefit of a matching system, motivating many residents to remain at the same hospital where they had just completed their 2-year residency. Specialty training typically lasted anywhere from four to 6 years, depending on the specialty and location. Before 2004, specialty training was provided for residents immediately after they graduated from medical school.

Second, the new matching system allowed medical school graduates to select a specialty training site designated by the MHLW, which included both university- and non-university-affiliated hospitals, as well as public and private institutions [2]. Before 2004, a majority of new graduates entered their specialty training program at the university hospital associated with the medical school they attended.

The revised matching system widened disparities between urban and rural areas for the following two reasons.

First, as prescribed by the 2004 reform, medical graduates would participate in a national matching system, while conveying personal residency location preferences [2]. Before 2004, physicians were dispatched to non-university hospitals by the department of their specialization at the university hospital, without undue consideration of individual choice.

Second, through the new matching system for the rotation residency training, graduates were now selecting independent urban hospitals rather than being assigned to those affiliated with a university [3, 4]. Because many graduates remained with the same institution to complete the specialty residency, decreases in incoming physicians at university hospitals resulted. Charged with dispatching physicians to other affiliated hospitals often located in rural and underserved areas, university hospitals found it increasingly difficult to serve these regions. Physicians working in rural areas returned to university hospitals to fill the void that the new training program created [5], with the Japan Medical Association Research Institute reporting an almost 60% decrease in the dispatch of physicians to other medical institutions by university hospitals [6].

Yet another issue arose as Japan strove to improve medical residency programs. In Japan, the general residency matching program focused on available positions, with hospitals in large cities capable of providing, by far, the most positions, and rural hospitals offering few, if any. In 2008, the Japanese government instituted the policy of *chiikiwaku*, a regional quota system, offering scholarship incentives to local prefectural residents to study medicine and return to practice in the prefecture for a minimum of 6 years. Currently, up to 17% of medical school entrants are participating in this quota system. This voluntary program was designed to aid in controlling distribution of physicians throughout the country, and yet, other factors influenced distribution of residents, as the choice of non-university hospitals in each region hindered transfer to rural regions. Moreover, there was no provision to control the number of residents entering into particular specialties, as long as a chosen hospital could accommodate them. As a result, residents were relatively free to choose their specialty, regardless of pressing needs for impacted specialties.

Subsequently, Japanese media reported that new physicians observed quality of life issues during residency training, preferring not to choose pediatrics or obstetrics, which can have higher risks of lawsuits and often oppressive working conditions. Instead, media reported that residents gravitated towards specialties such as dermatology or ophthalmology, where perceived workloads and lawsuit risks were not excessive because they treat mainly non-life-threatening conditions and maintain a more conventional work schedule. Without a cap on number of residents choosing a particular specialty, the media conjectured an expected increase in both ophthalmologist and dermatologist supply at the expense of *other physicians*, especially after the 2004 reform. The Japan Ophthalmologists Association issued a letter of protest, stating that media reports were not based on scientific evidence [7].

In an earlier paper, we explored effects of Japan’s 2004 postgraduate training reform on physician supply from two perspectives. First, we focused on pediatrician supply, as the most vulnerable population is children [8]. We clarified that disparities in pediatrician supply gradually declined until launch of the training reform, increasing afterwards. Also, the study showed pediatricians supply increased in urban centers and areas with higher *socioeconomic status* (SES) after 2004, while no such associations were found before 2004. Second, since the reform was perceived to affect physicians from hospitals more than clinics, we disaggregated hospital clinicians from clinic-based physicians to explore the effect on physician supply [9]. The study determined that while disparities in clinic-based physician supply did not change after the reform launch, disparities in hospital-based physician supply increased, fueling an increase in geographical inequality. The study also demonstrated that physicians working at the hospitals tended to move to urban centers and communities with higher SES after 2004, whereas no such tendency was found before 2004. Interestingly, clinic-based physicians tended to move to urban centers and communities with higher SES both before and after 2004, having been able to decide their preferred practice location even before launch of the 2004 program, while the physicians at hospitals gained such freedom only after the launch. The study indicated that physicians will move to urban areas and areas with higher SES when practice location choice is left to individual freedom.

While other published quantitative analyses of the 2004 program’s impact on physician distribution have previously failed to corroborate these views [10–13], results of our studies were more in line with the public’s perception of physician disparities. So, we employed methods similar to the ones we developed in our previous studies, and evaluated changes in ophthalmologist supply, which the Japanese media specifically named as the specialty with an increased supply after 2004, relative to *other physicians*. We also explored the influence of local demand and community level factors on ophthalmologist distribution. The underlying implication of our study is that due to the more predictable and normalized schedule of an ophthalmologist, sources such as the news media could interpret the specialty as best reflecting a higher quality of life for a Japanese physician.

## Methods

### Unit of analysis

We conducted an ecological analysis of ophthalmologist supply and distribution. Three-fourths of Japan consists of difficult terrain, including steep mountainous regions and forests, with less than 5% of the land area serving as residential. As a result, Japan’s geography imposes challenges to providing all levels of healthcare to even some relatively large municipalities. For that reason, we chose the “secondary tier of medical care” (STM) as the main geographical unit of analysis. An STM is roughly comparable to a Hospital Service Area in the U.S., and defined as a unit within which patients should be able to complete general medical care. Geographical conditions and infrastructure, as well as average traffic conditions, are considered to establish a STM. A STM typically comprises several municipalities, which are roughly comparable to U.S. cities or greater metropolitan areas. During the study, Japan reorganized through a large-scale merging of municipalities, with total municipalities decreasing from 3232 to 1742 between 1998 and 2012. Upon analysis, that necessitated data for former smaller municipalities to be merged into the data of the new larger municipal boundaries. Japan has 47 prefectures, roughly equivalent to U.S. states, each encompassing between four and ten STMs. STMs are independent administrative health service areas based on medical resources, transportation, and geography, and are less likely to suffer local spillovers than municipalities or counties [10, 14–19]. STM boundaries from 2012 (*n* = 346) were used for our study, as the total number of STMs did not change significantly between 1998 and 2012, in spite of some redrawn borders. Data applied at the prefecture level included meteorological values, which were not uniformly available at the STM level.

### Data

The difference in number of ophthalmologists between 1998 and 2002 (*pre-period*, i.e., before introduction of the 2004 program) and 2006–2010 (*post-period*, i.e., after the 2004 launch) were our main dependent variables of interest. Predictors with negative estimates denote a decrease in numbers of ophthalmologists, and positive estimates denote an increase.

As a comparison, analysis of the physician supply excluding ophthalmologists (i.e., *other physicians*) was explored. Difference in number of *other physicians* between 1998 and 2002 was used for *pre-period*, with the number of medical residents included in the physician count for each chosen specialty. However, the survey of Physicians, Dentists and Pharmacologists [20], used as our data source, was revised in 2006, and the new category “Residents” was added. Since residents were now rotated through different specialties with the 2006 changes, physician numbers for each specialty in the post-period no longer included residents. Differences in physician numbers, minus ophthalmologists and residents, were used as *other physicians* for post-period as a result.

Measures of need, and *community factors* as an indicator of residential quality and other regional advantages such as further access to professional development, were the two main predictors of interest for changes in physician supply, with *community factors* generally cited by literature as a major factor in physicians’ practice location preference [21–24]. Ophthalmologist density (number of ophthalmologists per 1000 population) and *other physician* density (number of *other physicians* per 1000 population) provided the measure of need. The rationale behind this was that if the number of ophthalmologists or *other physicians* per 1000 population was lower, there would be more need because of the disparities between areas with higher Ophthalmologist or *other physician* densities.

Because urban/rural status can be considered an indicator of *community factors*, we divided municipalities into five classifications, based on the metropolitan area codes defined by the Ministry of Internal Affairs and Communications and revised every 5 years with Japan’s national census: [25, 26] 1) central cities of major (greater) metropolitan areas, 2) central cities of metropolitan areas, 3) surrounding municipalities of major metropolitan cities (similar to US suburbs), 4) surrounding municipalities of metropolitan cities (similar to US suburbs), and 5) other municipalities.

Aligning to census years, we used urban/rural classifications for year 2000 for *pre-period* (1998–2002) and year 2005 for *post-period* (2006–2010). We combined major metropolitan and metropolitan areas into one category, given that there were only five central cities among 1742 municipalities in 2000 and six in 2005, and suburban regions into another, reducing categories into three groups: 1) STMs defined as urban centers that included central cities of major metropolitan areas, and metropolitan areas (*n* = 26 pre-period and *n* = 28 post-period); 2) STMs defined as suburban areas that included surrounding municipalities of urban centers (*n* = 127 pre-period and *n* = 130 post-period); and 3) STMs defined as rural or “other” municipalities (*n* = 193 pre-period and *n* = 188 post-period) that included all other areas not a part of central cities of major metropolitan areas, metropolitan areas, or surrounding municipalities of urban centers.

Beyond urban/rural status as a proxy for *community factors*, we also developed a composite area-based index of socioeconomic status (SES), representing demographic factors such as education, occupation, and income. SES was derived from a factor analysis of the percentage of white-collar workers and those with college-level education in the local population, as well as unemployment rates and per capita income. Using principal component analysis with varimax rotation, these factor scores were used to construct a composite index for each aspect of socioeconomic status [27].

Model variables are described in Table 1. Annual average temperature and humidity are available only at the prefecture level. Moreover, contact with well-regarded mentors are important to physicians in terms of training and future advancement, with published literature suggesting that physician practice location is influenced by professional interactions [21–24].

**Table 1:** Variables selected in the models

Variables	Explanation
Measures of Needs	
Ophthalmologist density	Number of ophthalmologists per 1000 population
Density of physicians other than ophthalmologists^e^	Number of physicians other than ophthalmologists per 1000 population
Density of physicians other than ophthalmologists and residents ^f^	Number of physicians other than ophthalmologists and residents per 1000 population Number of residents were not available prior to 2004
Measures of Community Factors	
Urban/rural status 1) urban centers 2) suburban areas 3) rural areas	Metropolitan area code defined by the Ministry of Internal Affairs and Communications
Per capita income	
Percent of the population with a university-level education	As a proxy for educational level in the community
Unemployment rate	Number of unemployed individuals per number of all individuals currently in the labor force (workforce)
Percent of white-collar workers	Number of professionals, technical workers, managers, and administrators per number of workforce
Primary school students per number of primary schools	As a proxy for children’s’ educational opportunities
Crime rate	Number of crimes per total population as a proxy for neighborhood safety
Temperature	As a proxy for climate discomfort. The discomfort index was calculated by using temperature and humidity and used in the model.
Humidity	
Measures of Professional interactions	
Hospital beds per 1000 population	
The presence or absence of medical schools	As a proxy for continuing education

### Data sources

All study data came from publicly-available secondary datasets, including factors previously shown to be associated with physician supply [21–24]. To calculate physician-to-population ratios, local population numbers were extracted from the Basic Resident Registers [28] and physician totals were obtained from the Survey of Physicians, Dentists, and Pharmacologists, which the MHLW conducts every 2 years. Under the Medical Practitioners Law, all licensed physicians must complete this survey, including medical specialty and employment address information [20].

The 2000 and 2005 Japanese Census data provided the following four variables [25, 26, 29]: 1) Metropolitan area codes; 2) percentage of population with a college-level education; 3) unemployment rate; and 4) percentage of white-collar workers. The *Regional Statistics by Municipalities,* produced by the Ministry of Internal Affairs and Communications (MIAC), provided [30]: 1) per capita income; 2) number of hospital beds; 3) number of primary schools; 4) number of primary school students; and 5) crime rates (number of crimes per 1000 population). Unemployment rates and percentage of white-collar workers were calculated using the mean of 1995 and 2000 data, applied to the 1998–2002 time period. Corresponding data from 2005 were applied to the analysis of the 2006–2010 time period. Because the percentage of population with college-level education is collected every 10 years, and 1990 data was no longer publicly available, data from 2000 was applied to the 1998–2002 time period. The mean of 2000 and 2010 data was applied to the 2006–2010 time period.

The discomfort index, developed by what is currently the National Weather Service to assess average climate considerations, was widely used as a proxy for climate discomfort in previous studies [31, 32]. Calculated by using temperature and humidity, the index was factored into potential physician location preferences as another indicator of community factors. *Regional Statistics by Prefectures*, produced by MIAC [33] provided temperature and humidity data for the model, which was available only at the prefecture level.

### Statistical analysis

The aggregate level change in ophthalmologists and *other physicians* from 1998 to 2012 at the national level, additionally stratified by urban/rural status, was explored. For the periods 1998–2002 (*pre-period*) and 2006–2010 (*post-period*), descriptive statistics of all continuous variables were calculated as means, with standard deviations and 95% confidence intervals (CIs). Mean equality tests were performed, examining the statistical significance of observed differences.

For both *pre-* and *post-period*, ordinary least-squares regression models were used to analyze changes in STM-level supplies of ophthalmologists and *other physicians*, evaluated as a function of STM-level baseline factors defined as 1998 conditions for *pre-period* and 2006 conditions for *post-period.* The 2006 survey revision, creating a separate category for all residents, necessitated defining 2006 data as a separate baseline. A coefficient equality test in regressions was performed, examining significant differences in coefficients between *pre-* and the *post-period.*

Last, we investigated changes in access to ophthalmologists over time between the best-supplied top 10% of STMs and the least-supplied bottom 10% of STMs for the period 1998–2012.

A two-tailed *P* value of less than .05 was considered statistically significant. All analyses were performed using SAS 9.4 Software (SAS Institute, Inc., Cary, NC, USA).

## Results

The aggregate level change in ophthalmologist and *other physician* supplies from 1998 to 2012 at the national level is shown in Table 2, additionally stratified by urban/rural status. In *pre-period*, relative change in ophthalmologist supply was slightly higher than in *other physician* supply (ophthalmologist 9.15% versus *other physician* 8.29%), whereas, in *post-period*, relative change in ophthalmologist supply was less than *other physician* supply (ophthalmologist 3.83% compared to *other physician* 10.23%).

**Table 2:** The aggregate level change in supply of ophthalmologists and other physicians at the national level

	1998	2000	2002	2004	2006	2008	2010	2012
National								
All other physicians^a^ (A)	225,525	231,141	237,126	244,216	236,776	244,724	253,082	260,997
Ophthalmologists (B)	11,408	12,060	12,448	12,452	12,362	12,627	12,797	12,835
Total population (in thousands) (C)	125.57	126.07	126.07	126.82	127.06	127.07	127.06	126.66
All physician density (A/C)	1.80	1.83	1.88	1.93	1.86	1.93	1.99	2.06
Ophthalmologists Density (B/C)	0.09	0.10	0.10	0.108	0.10	0.10	0.10	0.10
Relative change in number of all physicians	baseline	2.49%	5.14%	8.29%	baseline	3.36%	6.89%	10.23%
Relative change in number of ophthalmologists	baseline	5.72%	9.12%	9.15%	baseline	2.14%	3.52%	3.83%
Urban Center								
All other physicians^a^ (A)	84,831	86,893	88,551	91,362	73,126	74,299	75,461	76,695
Ophthalmologists (B)	4490	4731	4790	4783	3177	3196	3219	3240
Total population (in thousands) (C)	33.64	33.91	33.91	34.58	39.27	38.84	38.45	37.99
All other physician density (A/C)	2.52	2.56	2.61	2.64	1.86	1.91	1.96	2.02
Ophthalmologists Density (B/C)	0.13	0.14	0.14	0.14	0.08	0.08	0.08	0.09
Relative change in number of all other physicians	baseline	2.43%	4.39%	7.70%	baseline	1.60%	3.19%	4.88%
Relative change in number of ophthalmologists	baseline	5.37%	6.68%	6.53%	baseline	0.60%	1.32%	1.98%
Suburban								
All other physicians^a^ (A)	80,961	83,419	86,286	89,508	89,362	91,912	94,903	97,656
Ophthalmologists (B)	3942	4180	4387	4424	4552	4666	4736	4666
Total population (in thousands) (C)	51.92	52.25	52.25	52.70	53.89	54.01	54.10	54.00
All physician density (A/C)	1.56	1.60	1.65	1.70	1.66	1.70	1.75	1.81
Ophthalmologists Density (B/C)	0.08	0.08	0.08	0.08	0.08	0.09	0.09	0.09
Relative change in number of all other physicians	baseline	3.04%	6.58%	10.56%	baseline	2.85%	6.20%	9.28%
Relative change in number of ophthalmologists	baseline	6.04%	11.29%	12.23%	baseline	2.50%	4.04%	2.50%
Other								
All other physicians^a^ (A)	71,141	72,889	74,737	75,798	68,759	69,750	70,865	72,011
Ophthalmologists (B)	2976	3149	3271	3245	2952	2970	2985	3014
Total population (in thousands) (C)	40.01	39.91	39.91	39.55	36.85	36.43	36.05	35.60
All other physician density (A/C)	1.78	1.83	1.87	1.92	1.87	1.92	1.97	2.02
Ophthalmologists Density (B/C)	0.07	0.08	0.08	0.08	0.08	0.08	0.08	0.09
Relative change in number of all other physicians	baseline	2.46%	5.05%	6.55%	baseline	1.44%	3.06%	4.73%
Relative change in number of ophthalmologists	baseline	5.81%	9.91%	9.04%	baseline	0.61%	1.12%	2.10%

^a^all physicians other than ophthalmologists before 2004, and all physicians other than ophthalmologists and residents after 2006

Descriptive statistics of dependent and independent variables for *pre-period* and *post-period* are shown in Table 3, along with the *P* value resulting from testing the Equality of Means. It is worth noting that ophthalmologist density did not significantly increase (*P* = .061), whereas other physician density did increase significantly (*P* < .001).

**Table 3:** Descriptive statistics of all dependent and independent variables, the secondary tier of medical care (STM) as a unit of analysis

	1998–2002			2006–2010			*P* ^*c*^
	Mean	SD^a^	95% CIs^b^	Mean	SD^a^	95% CIs^b^	
Number of ophthalmologists	36.40	57.36	[32.91 to 39.90]	34.60	55.05	[31.25 to 37.95]	.47
Number of physicians other than ophthalmologists	749.60	1088.43	[683.31 to 815.89]	668.39	946.99	[610.72 to 726.07]	.07
Number of physicians other than ophthalmologists and residents^d^	NA	NA	NA	707.69	1013.78	[645.94 to 769.43]	NA
Ophthalmologists density^e^	0.078	0.043	[0.076 to 0.081]	0.075	0.044	[0.072 to 0.077]	.061
Density of physicians other than ophthalmologists ^f^	1.77	0.81	[1.72 to 1.82]	1.62	0.76	[1.58 to 1.67]	< .001
Density of physicians other than ophthalmologists and residents^d,g^	NA	NA	NA	1.69	0.74	[1.64 to 1.73]	NA
Total population(in thousands)	364.20	399.50	[334.40 to 394.00]	367.20	417.70	[336.00 to 398.40]	.89
Per capita income (in thousands)^h^	11.75	3.31	[11.51 to 12]	11.36	3.15	[11.12 to 11.59]	.023
Percent of the population with a college-level education	10.81	5.25	[10.26 to 11.37]	11.92	5.39	[11.35 to 12.49]	.006
Unemployment rate	4.06	1.19	[3.93 to 4.18]	5.78	1.50	[5.63 to 5.94]	< .001
Percent of white-collar workers	14.42	2.40	[14.17 to 14.68]	13.96	2.25	[13.73 to 14.2]	.010
SES composite Index^i^	− 0.02	1.02	[−0.13 to 0.09]	0.02	0.98	[−0.08 to 0.12]	.59
Number of primary students/school	281.20	133.00	[271.3 to 291.1]	274.00	138.80	[263.7 to 284.4]	.33
Crime rate	1.47	0.73	[1.42 to 1.52]	1.10	0.53	[1.06 to 1.14]	< .001
Temperature (°C)	15.82	2.53	[15.3 to 16.34]	15.59	2.36	[15.10 to 16.07]	.52
Humidity (%)	70.28	4.70	[69.31 to 71.25]	69.44	4.36	[68.54 to 70.33]	.21
Discomfort Index^j^	60.05	3.87	[59.25 to 60.84]	59.68	3.58	[58.94 to 60.41]	.50
Hospital beds per 1000 population	13.89	4.75	[13.54 to 14.24]	13.89	4.62	[13.55 to 14.24]	.99

^a^Standard Deviation

^b^Confidence Intervals

^c^*p-value* of mean equality test

^d^Number of residents were unavailable prior to 2004

^e^Number of ophthalmologists per 1000 population

^f^Number of physicians other than ophthalmologists per 1000 population

^g^Number of physicians other than ophthalmologists and residents per 1000 population

^h^Japanese yen was converted into US$ using the rate that applied in March 2013 of approximately 95 Japanese yen per US$

^i^A composite index of socioeconomic indicators created from the percent of the population with a college-level education, percent of white-collar workers, the unemployment rate, and per capita income

^j^Calculated by using temperature and humidity

Table 4 reveals results from multivariate ordinary least-squares regression models, with main predictors of interest controlled by all other factors shown in Table 1 for ophthalmologist supply. The change in ophthalmologist supply was inversely correlated to baseline ophthalmologist density both in *pre-* and *post-period* (*P* < .001 in *pre-period* and *P* = .01 in *post-period*). In other words, areas with lower ophthalmologist density attracted more ophthalmologists, resulting in an overall decrease in supply inequalities among regions. After adjustment for all other variables, we estimated that each unit increase in ophthalmologist density (1 ophthalmologist per 1000 population) in 1998 was associated with an increase in the number of ophthalmologists of 60.34 in 2002, and each unit increase in 2006 with an increase of 42.94 in 2010. Estimates for urban centers and sub-urban areas were negative in *pre-period*, while positive in *post-period*, which resulted in a significant difference in the estimate between the two periods (*P* = .018 and *P* = .033).

**Table 4:** Results of multivariate regression models for ophthalmologists^a^and physicians other than ophthalmologists^a^

Parameter	1998–2002					2006–2010					*P* of coefficient equality test
	Estimate	SE^b^	95%CI^c^		*P* value	Estimate	SE^b^	95%CI^c^		*P*	
For ophthalmologist											
Opt density^d^	− 60.34	14.16	[−88.09	−32.59]	< .001	−42.94	16.55	[− 75.38	− 10.50]	0.01	0.43
Other physician density^e^	1.95	0.88	[0.22	3.68]	0.027	5.41	1.06	[3.33 7.50]		<. 001	0.013
Urban center	−1.92	1.32	[−4.51	0.67]	0.15	2.87	1.51	[−0.09	5.83]	0.06	0.018
Suburban	−1.24	0.58	[−2.39	−0.10]	0.034	0.73	0.71	[−0.66	2.13]	0.3	0.033
others	reference					reference					NA
SES Index^f^	1.04	0.41	[0.23	1.84]	0.012	0.18	0.5	[−0.79	1.15]	0.72	0.18
For physicians other than ophthalmologists											
Opt density^d^	− 143.69	126.77	− 392.15	104.77	0.26	233.81	159.71	−79.22	546.84	0.14	0.065
Other physician density^e^	5.27	7.89	− 10.2	20.75	0.5	51.26	10.27	31.14	71.39	<. 001	<. 001
Urban center	−21.78	11.82	−44.95	1.4	0.07	72.66	14.58	44.08	101.24	<. 001	<. 001
Suburban	−7.14	5.23	−17.4	3.12	0.17	−5.04	6.89	−18.54	8.45	0.46	0.81
others	reference					reference					NA
SES Index^f^	7.51	3.68	0.29	14.73	0.041	10.89	4.78	1.52	20.25	0.023	0.58

^a^The models included the control variables: number of primary school students per number of primary schools, crime rate, discomfort index calculated by temperature and humidity, hospital beds per 1000 population, and the presence or absence of medical schools

^b^Standard Error

^c^Confidence Intervals

^d^Ratio of number of ophthalmologists to 1000 population

^e^Ratio of number of all physicians other than ophthalmologists to 1000 population

^f^SES composite index was created from the percent of the population with a college-level education, percent of white-collar workers, the unemployment rate, and per capita income

Results in the main predictors of interest from multivariate regression models are shown in Table 4, as controlled by all other factors for *other physician* supply shown in Table 1. Change in *other physician* supply was not positively correlated to baseline *other physician* density in the *pre-period,* but it was positively correlated in the *post-period.* In other words, in the post-2004 period, areas with already high physician supply attracted even more physicians. While the change in *other physician* supply was not correlated to choice of urban centers in the *pre-period* (*P* = .07), it was positively correlated in the *post-period* (*P* < .001).

Table 5 shows ophthalmologist supply for the best-supplied top 10% of STMs, as well as the least-supplied bottom 10% of STMs in 2-year increments from 1998 to 2012. Ophthalmologist density gradually increased both in the top and bottom 10% of STMs over the period. Although inequalities remained large, with the best-served areas benefiting from coverage levels averaging five times higher than least-served areas, coverage inequalities gradually declined to some extent from 1998 to 2012.

**Table 5:** Ophthalmologist supply of the top 10% and bottom 10% of the secondary tiers of medical care (STMs)

	1998	2000	2002	2004	2006	2008	2010	2012
Top 10% (*n* = 35)								
# of Ophthalmologists^a^	4321	4538	4680	4555	4381	4430	4342	4635
Total population	27,674,836	28,101,936	28,811,684	28,093,204	27,631,756	27,408,660	26,493,008	28,386,764
Density^b^ (A)	0.16	0.16	0.16	0.16	0.16	0.16	0.16	0.16
Bottom 10% (n = 35)								
# of Ophthalmologists^a^	86	100	108	109	102	99	126	101
Total population	3,395,909	3,544,457	3,539,238	3,618,177	3,430,603	3,333,489	3,922,762	3,195,039
Density^b^(B)	0.03	0.03	0.03	0.03	0.03	0.03	0.03	0.03
Ratio^c^(A/B)	6.17	5.72	5.32	5.38	5.33	5.44	5.10	5.17
Difference^d^(A-B)	0.13	0.13	0.13	0.13	0.13	0.13	0.13	0.13

^a^Number of ophthalmologists

^b^Number of ophthalmologists per population

^c^Ratio in ophthalmologist density

^d^Difference in ophthalmologist density

## Discussion

The current study explored the impact of the 2004 national physician training reform, determining whether ophthalmologist supply increased at a higher rate after 2004, as some Japanese media had reported [7]. We found two points that contradict the media reports. First, relative increase in ophthalmologist supply from 2006 to 2010 was 3.83%, while more than 10% for *other physicians*. The increase in ophthalmologist supply before the launch of the reform was greater than increases in *other physician* supply. However, after the reform, the increase in ophthalmologist supply was actually smaller than increases in *other physician* supply. Second, although *other physician* density significantly increased after the launch of the reform, ophthalmologist density did not change between the two periods.

Interestingly, we clarified that the geographical inequality of ophthalmologist supply coverage decreased over our study period, while *other physician* coverage increased in inequality. More specifically, this conclusion was prompted by the following two results. First, ophthalmologists tended to move to locations with lower ophthalmologist densities both before and after 2004, while other physicians tended to move to locations with higher physician densities after 2004, but not before. Second, inequality in ophthalmologist coverage among the best-supplied top 10% of STMs and the least-supplied bottom 10% of STMs constantly decreased throughout the study period. In contrast, Sakai et al... [8] reported that the inequality in pediatrician supply declined from 1998 to 2002, while increasing post-2004-reform. Shimomura et al [34] also reported that the geographical inequality of ophthalmologist supply coverage was large compared to other specialties, but that these disparities were slowly receding, which is consistent with our report.

Until 2004, new graduates from Japan’s medical schools typically entered specialty training at university hospitals, and then were dispatched to affiliated hospitals. The launch of Japan’s matching system in 2004 changed physician placement. Rather than choosing university hospitals for residency training, young physicians tended to stay at the hospitals where they received residency training. However, in the case of those receiving board-certified training as ophthalmologists, physicians were assigned to hospitals with more than 6 board-certified ophthalmologists, as designated by the Japanese Ophthalmological Society. These were customarily university hospitals, and included only a limited number of exceptional major hospitals [35]. Consequently, even after 2004, ophthalmologists were typically concentrated in university hospitals, and therefore, more likely to be dispatched to affiliated hospitals. It is also interesting that other physicians tended to concentrate in urban centers, while ophthalmologists were distributed into both suburban and urban centers.

Having reported increased disparities in physician distribution after the 2004 reform, we suggested the need for new placement schemes for better equity in healthcare access [8, 9]. It is noteworthy that, post-2004, ophthalmologist distribution revealed greater equality of access, because with training still modeled on the old system, ophthalmologists have been affiliated with and dispatched by university hospitals. This study showed that the system to obtain a board-certified specialization for ophthalmologists, as implemented by the Japanese Ophthalmological Society and Japan Ophthalmologists Association, could be a model for placement of other physicians If other medical specialties have retained, or intend to retain, the pre-2004 board certification system, and as a result, the specialties show similar patterns of distribution to ophthalmologists, this would amount to the stronger evidence that the board certification system can help counter disparities.

Another interesting finding is that the rate of increase in the number of ophthalmologist is very low compared to that in other physicians, which contradicts the media’s aforementioned statements. Because there was no formal policy cap on residency specialties, numbers directly reflect specializations chosen by medical graduates. Shimomura et al. [34] also reported that the number of young physicians who entered specialty training in ophthalmology was reduced by half in 2011, compared to 2001, which is in agreement with our results.

In 2017, Japan is expected to launch a new board-certified specialty system that is standardized throughout the medical specialties. Details of the new system have not been published yet, but further observation is warranted, in case the new system negatively impacts the distribution of ophthalmologists.

The Japanese Ophthalmological Society [7] noted that prevalence of ophthalmic diseases, including vision disorder, is likely to increase due to the aging of society. Given that vision disorders greatly affect seniors’ quality of life, an equitable supply of ophthalmologic care is crucial, as with many other specialties. Our research indicated that while the distribution of ophthalmologists was less impacted by the 2004 policy change in residency training, the distribution of *other physicians* did not fare as well. In lieu of the oversight that Ophthalmologists still receive in their profession, quality of life issues, such as community factors, may create further disparities in both physician distribution and accessibility to needed specialties. More research and creative policies are needed to merge the needs of patients with the important quality of life issues experienced in the physician profession.

Limitations in this study include, first, the lack of publicly-available data to indicate whether the physician’s work status is full-time or part-time. Additionally, given the fact that overall headcounts were used to count the number of physicians, overestimation could result from physicians working in more than one location.

Second, we ignored the distinction between hospital-based clinicians and smaller-clinic-based physicians (i.e. primary care clinics with fewer than 20 beds) due to the unavailability of the data. We have clarified that the spatial distribution for clinic-based physicians was not affected by the new scheme as much as hospital-based physicians, because the 2004 reforms affected resident training programs that take place primarily in training hospitals designated by the MHLW, as opposed to physicians’ practice locations in small clinics [8, 9]. Additionally, the percentages of clinic-based ophthalmologists (between 50 and 60%) during the study periods were much higher than percentages of all other physicians who were clinic-based (about 40%) [36]. This might affect the results shown in this study. Detailed analyses stratified by clinic-based and hospital-based ophthalmologists should be conducted if data are available through a non-publicly-available dataset.

Third, as one of the measures of need for physician supply, previous studies included child mortality for pediatricians [8] and age-adjusted mortality for all physicians [9]. However, only physician density is applicable in this study, with ophthalmologist and other physician densities as measures of need. We did not consider the prevalence of blindness a suitable proxy as a health need indicator, because ophthalmologists typically have a much greater role in the prevention of blindness than management of the condition.

Fourth, it was unfortunate that annual average temperature and humidity were available only at the prefecture level. Nonetheless, we still included them in the regression models as a proxy.

Fifth, there was no standardized urban/rural definition for STMs. In our definition, urban STMs could include rural municipalities. However, rural STMs do not include urban municipalities, and thus, the results of this study could be a proxy for the movement of physicians between urban and rural areas.

Sixth, it is clearly possible that other changes, such as the establishment of new health facilities, could be correlated with changes in ophthalmologist supply, and lead to confounding bias in our analysis. Moreover, parental childcare and maternity leave laws have been added or strengthened to keep parents in the workforce within the timeframe of our study. ([37]) One new policy was the revised childcare leave law in 2010, with up to a year of leave for mother or father. Research showed that women were avoiding specializations that interfered most with raising a child, such as surgery or on-call positions, with up to 75% dropping out once they had a child. Ophthalmology, and other similar specialties like dermatology, would be considered more suitable positions for mothers, since their hours would occur mostly during normal working shifts. So, better childcare laws could have the effect of encouraging women to pick other specialties, which might account for the rise in other specialties during this time.

Last, to attract physicians to rural, underserved areas, some local governments have developed strategies, such as public funding for higher salaries, that might also influence physician practice locations. Reported coefficients should reflect these effects, since omitting a variable in the model for this type of aid may underestimate policy effects on location choices. However, the ophthalmologist supply is less likely to be affected by this local policy, since they require very specific training and are likely to be affiliated to, and dispatched by, university hospitals.

## Conclusions

Although ophthalmologist supply increased between 1998 and 2012, it increased less than overall physician supply. Even after the launch of the 2004 postgraduate training reform, ophthalmologist supply increased at a lower rate than other physician supply. Ophthalmologists tended to move to places with lower ophthalmologist density, both before and after the launch of the 2004 postgraduate training reform, likely because the 2004 reform did not change the placement system substantially.

## Abbreviations

CIs: Confidence intervals
MHLW: Ministry of Health, Labor and Welfare
MIAC: Ministry of Internal Affairs and Communication
PGME: Postgraduate medical education
SES: Socioeconomic status
STM: Secondary tier of medical care
